# Considerations in Audio-Visual Interaction Models: An ERP Study of Music Perception by Musicians and Non-musicians

**DOI:** 10.3389/fpsyg.2020.594434

**Published:** 2021-01-20

**Authors:** Marzieh Sorati, Dawn M. Behne

**Affiliations:** Department of Psychology, Norwegian University of Science and Technology, Trondheim, Norway

**Keywords:** musicians and non-musicians, event-related potential (ERP), music perception, audiovisual perception, auditory perception

## Abstract

Previous research with speech and non-speech stimuli suggested that in audiovisual perception, visual information starting prior to the onset of corresponding sound can provide visual cues, and form a prediction about the upcoming auditory sound. This prediction leads to audiovisual (AV) interaction. Auditory and visual perception interact and induce suppression and speeding up of the early auditory event-related potentials (ERPs) such as N1 and P2. To investigate AV interaction, previous research examined N1 and P2 amplitudes and latencies in response to audio only (AO), video only (VO), audiovisual, and control (CO) stimuli, and compared AV with auditory perception based on four AV interaction models (AV vs. AO+VO, AV-VO vs. AO, AV-VO vs. AO-CO, AV vs. AO). The current study addresses how different models of AV interaction express N1 and P2 suppression in music perception. Furthermore, the current study took one step further and examined whether previous musical experience, which can potentially lead to higher N1 and P2 amplitudes in auditory perception, influenced AV interaction in different models. Musicians and non-musicians were presented the recordings (AO, AV, VO) of a keyboard /C4/ key being played, as well as CO stimuli. Results showed that AV interaction models differ in their expression of N1 and P2 amplitude and latency suppression. The calculation of model (AV-VO vs. AO) and (AV-VO vs. AO-CO) has consequences for the resulting N1 and P2 difference waves. Furthermore, while musicians, compared to non-musicians, showed higher N1 amplitude in auditory perception, suppression of amplitudes and latencies for N1 and P2 was similar for the two groups across the AV models. Collectively, these results suggest that when visual cues from finger and hand movements predict the upcoming sound in AV music perception, suppression of early ERPs is similar for musicians and non-musicians. Notably, the calculation differences across models do not lead to the same pattern of results for N1 and P2, demonstrating that the four models are not interchangeable and are not directly comparable.

## 1. Introduction

In audiovisual (AV) perception, studies on speech have established that seeing a talker's face can facilitate reaction time and intelligibility, compared to unimodal auditory perception (Besle et al., 2004; Schwartz et al., 2004; Remez, 2005; Campbell, 2007; Paris et al., 2013; van Wassenhove, 2013; Karas et al., 2019). Similarly, in AV music perception, visual information from finger and hand movements while playing a musical instrument can enhance music perception (Maes et al., 2014). Visual information naturally starts before the auditory signal, and recent research shows that this visual information can also work as temporal (Paris et al., 2017), spatial (Senkowski et al., 2007a; Stekelenburg and Vroomen, 2012), and content (Doehrmann and Naumer, 2008) cues that provide predictions about an upcoming sound.

Previous electrophysiological evidence indicates that when visual information predicts a corresponding sound in AV perception, auditory and visual perception interact. AV interaction leads to a suppression in early event-related potentials (ERPs) such as N1 and P2 amplitudes and latencies (e.g., Klucharev et al., 2003; Van Wassenhove et al., 2005; Stekelenburg and Vroomen, 2007). N1 and P2 are both auditory evoked responses and are sensitive to changes in the physical attributes of auditory stimuli (Näätänen and Winkler, 1999; Tremblay et al., 2006), however, they have different underlying processes in AV perception (Van Wassenhove et al., 2005; Stekelenburg and Vroomen, 2007, 2012; Arnal et al., 2009; Paris et al., 2016a,b, 2017). N1, a negative component occurring around 100 ms after the stimulus onset, is sensitive to general attributes of the stimuli such as predictability of the upcoming sound based on the visual cues (Arnal et al., 2009; Paris et al., 2016a, 2017), spatial information (Stekelenburg and Vroomen, 2012), and temporal information (e.g., Senkowski et al., 2007b; Paris et al., 2017). For example, one study (Libesman et al., 2020) showed that in response to AV stimulus such as clapping hands, visual predictive information can affect the sensitivity of the auditory N1 amplitude to sound pressure level. Furthermore, P2, a positive component occurring around 200 ms after the stimulus onset, is sensitive to the content congruency and the integration between the visual information and perceived auditory signal (Van Wassenhove et al., 2005; Arnal et al., 2009; Paris et al., 2016b). In sum, in AV perception, while both N1 and P2 show AV interaction, N1 is more sensitive to the predictiveness of the visual cues, and P2 is more sensitive to the integration of auditory and visual information (e.g., Paris et al., 2016a, 2017).

Previous research has shown that suppression of N1 and P2 amplitudes and latencies in AV perception is not limited to speech (Oray et al., 2002; Stekelenburg and Vroomen, 2007, 2012; Paris et al., 2016a, 2017). Stekelenburg and Vroomen (2007) showed that AV interaction at N1 and P2 can be observed with non-speech stimuli, such as clapping hands. AV interaction in response to non-speech, as well as speech, can occur at N1 as long as the visual cues lead to a prediction of what and when the auditory sound is coming (Paris et al., 2017).

Other research has shown that AV interaction does not always lead to suppression in N1 and/or P2 amplitudes and latencies (e.g., Oray et al., 2002; Miki et al., 2004; Alsius et al., 2014; Baart, 2016; Paris et al., 2017). A meta-analysis of 20 different AV perception studies with speech stimuli (/ba/) (Baart, 2016) suggested that variability in N1 and P2 results across different studies may be dependent on factors such as experimental task and design. Moreover, Sorati and Behne (2019) showed that previous AV experience such as musical training can affect N1 suppression. That is to say, in response to a speech syllable /ba/, musicians showed more N1 amplitude suppression in AV perception compared to the auditory condition, while non-musicians did not show this pattern. Regardless of the musical background, P2 amplitude and latency were lower in AV perception, compared to auditory perception. These results suggest that a lack of suppression in N1 and/or P2 amplitudes and latencies in some studies might be dependent on factors such as experimental design and the participants' previous AV experience.

To examine the interaction of auditory and visual perception in electrophysiological studies, quantitative designs have been developed based on EEG signals evoked in response to audio only (AO), video only (VO), and AV stimuli. In one such design, illustrated in model (1) (Besle et al., 2009), AV interaction was estimated based on N1 and P2 amplitudes and latencies evoked by AV stimuli compared with a summation of evoked AO and VO signals. The underlying approach to AV interaction in model (1), which is also known as the “additive model” (e.g., Besle et al., 2004), is that if auditory and visual information were processed independently in AV perception, the neural activity induced by the AV stimulus should equal the sum of the responses separately elicited by the AO and VO stimuli. Any differences, superadditivity or subadditivity, in N1 and P2 amplitudes and latencies between signals evoked by AV stimuli compared with the summation of responses evoked by AO and VO stimuli, should be attributed to AV interaction between processing of auditory and visual information in AV perception (Besle et al., 2004, 2009; Talsma and Woldorff, 2005; Van Wassenhove et al., 2005; Pilling, 2009; Giard and Besle, 2010).

(1) $$\begin{array}{r} \text{AV }\text{vs. }\text{AO+VO} \end{array}$$

As a variation of the additive model approach for AV interaction, other studies (Arnal et al., 2009; Stekelenburg and Vroomen, 2012; Alsius et al., 2014; Baart et al., 2014; Paris et al., 2016a, 2017) have applied model (2) in which the difference waveform resulting from subtracting the VO from the AV waveform is compared with the AO waveform. While model (1) and (2) have a similar underlying additive approach, model (1) evaluates the relationship between the evoked signals in response to two AV conditions, AV and AO+VO, whereas model (2) evaluates the relationship between two auditory conditions, AO and AV-VO. Baart (2016) examined 20 different experiments on AV interaction with speech stimuli applying model (2), and results suggested that despite variations in suppression of N1 and P2 amplitudes and latencies, on average they were lower in AV-VO compared to the AO condition, a pattern of results which is similar to observations applying model (1) (e.g., Besle et al., 2004).

(2) $$\begin{array}{r} \text{AV-VO }\text{vs. }\text{AO} \end{array}$$

A possible caveat of model (2) led to the derivation of a new model from the additive approach. Stekelenburg and Vroomen (2007) argued that applying model (2) for examining AV interaction might lead to spurious AV interaction effects. For example, common activity, such as an anticipatory slow wave potential which may arise before each unimodal or bimodal stimulus and continue even after the stimulus onset, can be found in all conditions. While this common neural activity evoked in response to all stimuli types will also be present in the AO condition, they will be subtracted out in AV-VO. Therefore, to balance this spurious effect, as illustrated in model (3), they included an additional stimulus in the experiment: control (CO). The CO stimulus was a gray background with no sound which did not have any dynamic visual or auditory information. In this way, by subtracting CO from the AO waveform (AO-CO), the common neural activity evoked in response to AO will be subtracted out.

(3) $$\begin{array}{r} \text{AV-VO }\text{vs. }\text{AO-CO} \end{array}$$

Another model is based on a comparison between AV and AO. Although a direct comparison of AV and AO can lead to sensory confounds since these stimuli are in different modalities (Luck, 2014), based on a meta-analysis of studies applying model (2), Baart (2016) suggested that, first, there is no reason to assume additive models lead to the spurious AV interaction effects accounted for by model (3) since common neural activity present in response to all stimuli types disappear after high-pass filtering (>1 Hz) (Huhn et al., 2009). Baart (2016) further suggested that the calculated results for N1 and P2 amplitude and latency suppression based on model (2) is similar to model (4).

(4) $$\begin{array}{r} \text{AV }\text{vs. }\text{AO} \end{array}$$

Research has shown that previous AV experience, such as musical training, can enhance auditory processing through experience-based neural plasticity and enhance N1 and P2 amplitudes (Shahin et al., 2003, 2005; Kuriki et al., 2006; Baumann et al., 2008; Virtala et al., 2014; Maslennikova et al., 2015; Rigoulot et al., 2015; Sanju and Kumar, 2016, but also see Lütkenhöner et al., 2006). For example, Pantev et al. (2001) showed that musicians have higher early amplitudes in response to an auditory piano stimulus, compared to non-musicians. Studies (Shahin et al., 2003, 2005; Baumann et al., 2008; Maslennikova et al., 2015) have also shown that musicians have higher N1 and P2 amplitudes in response to both music and speech stimuli (Musacchia et al., 2008) compared to non-musicians.

Practicing a musical instrument can also enhance integration across sensory modalities (Zatorre et al., 2007; Strait and Kraus, 2014), and shape AV perception (Haslinger et al., 2005; Musacchia et al., 2008; Petrini et al., 2009a; Lee and Noppeney, 2011; Paraskevopoulos et al., 2012; Maes et al., 2014; Proverbio et al., 2016). For example, in behavioral and fMRI studies, Petrini et al. (2009a,b, 2011) have shown that drummers, compared to non-musicians, are more sensitive to AV synchrony in a drumming point-light task and showed decreased neural activity. Moreover, previous studies showed that musicians have increased intracerebral functional connectivity in theta, alpha and beta bands (e.g., Kühnis et al., 2014), which are essential for processing speech (Gisladottir et al., 2018), and music (Doelling et al., 2019). Sorati and Behne (2019) suggested that participants' previous musical experience can enhance N1 suppression and alpha desynchronization in response to AV speech. For AV music perception Sorati and Behne (2019) observed more beta suppression for musicians compared to non-musicians, despite no group difference for N1 or P2 based on model (2). Given the calculation differences across the four AV interaction models, the AV model applied to N1 and P2 measures may have consequences for evidence of AV interaction. Furthermore, based on model (2), Baart (2016) suggested that the N1 and P2 amplitudes and latencies in response to AO would correlate with those in AV perception. With this basis, an open question is whether musicians, who are broadly shown to have enhanced N1 and P2 amplitudes in response to auditory music perception (e.g., Shahin et al., 2003; Baumann et al., 2008) also show correlation between AO and AV-VO and, if so, whether they show more suppression of N1 and P2 amplitudes and latencies in AV music perception, compared to non-musicians.

The current study investigates differences between the four AV interaction models (1,2,3,4) by examining the suppression of N1 and P2 amplitudes and latencies in AV perception, compared to auditory perception. Previous studies indicate that when visual information predicts a corresponding sound in AV perception, auditory and visual perception interact. AV interaction leads to a suppression in early event potentials such as N1 and P2 (e.g., Van Wassenhove et al., 2005). Further research (e.g., Besle et al., 2004) suggested that any difference in N1 and P2 amplitudes and latencies between AV evoked waveforms compared to the mere summation of AO and VO waveforms can be attributed to AV interaction (model 1). Others (e.g., Paris et al., 2016a) compared AO waveforms and AV-VO waveforms (model 2). Moreover, Stekelenburg and Vroomen (2007) subtracted CO waveform from the AO waveform and then compared it to the AV-VO difference wave to avoid the spurious interaction effects due to common neural activity evoked in response to all of the stimuli (model 3). Finally, a meta-analysis for AV speech (Baart, 2016) suggested that even a mere comparison between AO and AV condition can also show the effect of AV interaction (model 4). All of these models originated from the additive model for AV perception and have been used vastly in previous research with speech and non-speech stimuli (e.g., Besle et al., 2004; Stekelenburg and Vroomen, 2007; Baart, 2016) as they express the same pattern of results (amplitude and latency suppression at N1 and P2 in AV perception) (e.g., Besle et al., 2004; Baart, 2016). However, the four models differ fundamentally in what they are comparing (sensory confounds); while model 1 is a comparison of two AV waveforms (AV and AO+VO), models 2 and 3 compare two auditory waveforms (e.g., AV-VO and AO), and model 4 is a comparison of an AV and an auditory waveform.

Baart (2016) further suggested a positive correlation between the magnitude of amplitudes and latencies in response to AO, and the size of amplitude and latency suppression at N1 and P2 in AV perception. While previous research (e.g., Shahin et al., 2005) showed that musicians, compared to non-musicians, have higher N1 and P2 amplitudes in response to auditory music, and also have enhanced N1 suppression in response to AV speech perception (Sorati and Behne, 2019), a remaining question is whether the potentially enhanced AO N1 and P2 amplitudes due to musical experience leads to more suppression of N1 and P2 amplitudes in AV perception? Thus, for the current study, each of the four AV interaction models will be investigated with musical experience as a case in point. First, musicians and non-musicians will be compared based on N1 and P2 amplitudes and latencies in response to AO music stimuli (C4 on keyboard). Then, suppression of N1 and P2 amplitudes and latencies for musicians and non-musicians will be compared based on each of the four AV interaction models (1, 2, 3, and 4), to examine if musicians' potentially higher AO N1 and P2 amplitudes lead to more suppression of N1 and P2 amplitudes and latencies in AV music perception.

## 2. Materials and Methods

This study was based on data recorded as part of a larger project to investigate the effect of musical experience on auditory and AV speech (Sorati and Behne, 2019) and music (Sorati and Behne, 2020) perception by comparing musicians and non-musicians' EEG data in response to audio, video and audio-video stimuli.

### 2.1. Participants

Forty adults (19 females) age 19–33 years (Table 1) participated in the experiment, of which 19 were musicians and 21 were non-musicians. Recordings from one additional male musician were excluded from the study due to technical issues. All participants were right-handed (based on a variant of the Edinburgh Handedness Inventory, Oldfield, 1971), had normal to corrected visual accuracy (Snellen test), had a pure tone auditory threshold of 15 dB HL for 250–4,000 Hz (British Society of Audiology, 2004), and Norwegian as their first language. None reported a history of neuropsychological disorders.

**Table 1:** Means and standard deviations (in parentheses) for participants based on information provided as questionnaire responses.

	**Age**	**Gender**	**Interest in music**	**Listening to music per week**	**Age starting an instrument**	**Years of musical experience**	**Practice per week**
Musicians	23 yrs (3 yrs)	9 females, 10 males	9(1)/10	19 hr (13 hr)	8 yrs (2 yrs)	16 yrs (5 hr)	15 hr (11 hr)
Non-musicians	23 yrs (3 yrs)	10 females, 11 males	5(2)/10	5 hr (5 hr)	-	<1 year	-

The musicians were students in Musicology or Music Performance studies at the Norwegian University of Science and Technology (NTNU) for which admission requires theoretical and practical evaluations. Musicians had advanced skills in at least one musical instrument (piano, keyboard, violin, guitar, saxophone, or percussion), and all reported practicing and regularly performing in public during the experiment's timeframe. Musicians with singing and dancing training were excluded from this study to isolate the musical experience to instrumental training (e.g., Karpati et al., 2017). Based on previous research examining the effect of musical background on auditory perception (Pantev et al., 2001; Kühnis et al., 2013), variation in musical instruments among the musicians is not expected to influence the results in the current study, since perception is enhanced by general effects of musical experience, rather than instrument-specific mechanisms. However, taking into consideration that instrument specific expertise is known to influence the underlying predictive processing among musicians (Heggli et al., 2019), musicians were recruited who had keyboard or piano in common as their main or secondary instrument. Musicians began formal musical training at the mean age of 8 and had been playing their musical instrument at least for 8 years. They reported their interest in music as, on average, 9 on a 10-point scale (1 was “not interesting at all” and 10 was “very interesting”) and none reported absolute pitch perception.

The non-musicians were also students at NTNU, however, none were students of music. Non-musicians had a maximum 1 year of weekly musical training which is mandatory in Norwegian elementary schools. They reported their interest in music as 5 out of 10 on average.

Before the experiment, all participants provided written consent according to the Norwegian Center for Research Data, and were given an *honorarium* after the experiment.

### 2.2. Stimuli Materials

**Figure 1:**
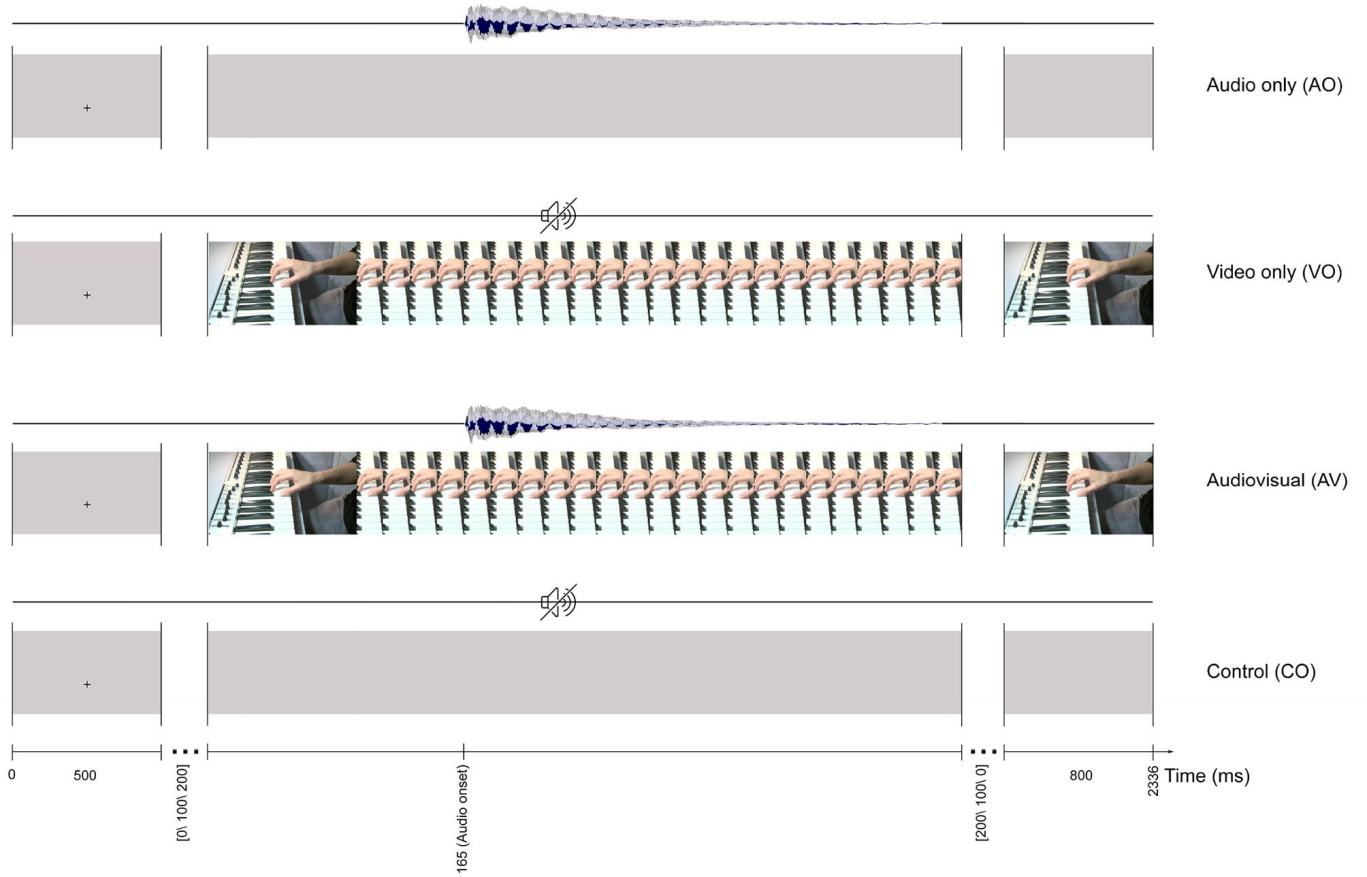
The trial timeline for four stimuli: audio only (AO), video only (VO), audiovisual (AV), and control (CO). All of the stimuli start with a 500 ms fixation cross and finish with an 800 ms still image of the last frame.

Stimulus materials were developed from an AV recording of an instrumentalist's right hand playing middle C4 on a keyboard, recorded in an IAC sound-attenuated studio (IAC acoustics, Hampshire, UK) in the NTNU Speech Laboratory at the Department of Psychology. First, using a Sony PMW-EX1R camera (30 fps) mounted on a tripod, an audio-video recording was made of an instrumentalist's right hand positioned on a keyboard (Evolution MK-449C, UK), with the left side of the thumb playing the middle C4 (261.6 Hz) key. The recorded audio track was replaced with a MIDI C4 note produced in GarageBand (10.0.3). Then, the AV material was exported in H.264 format with an MP4 container in Adobe Premiere Pro CS54.5. This formed the basis for the four stimuli illustrated in Figure 1: audio only (AO), which consisted of a 700 ms-long C4 audio track with a gray video background; video only (VO), which was the original video from the finger-hand movement on the keyboard playing the C4 key with no audio; audiovisual (AV), which was the audio with the video recording; and a control (CO), which had a gray background with no audio.

### 2.3. Procedure

The experiment was carried out in an IAC sound-attenuated studio at the NTNU Speech Laboratory. The studio was dimly lit during the experiment and to minimize head movements, participants positioned their head on a chinrest. Videos were presented at eye level on a 40" LCD flat panel display (Samsung SyncMaster 400DX-2) with a resolution of 1152 × 648. The display was approximately 190 cm away from the participant, so that the physical size of the video on the display was similar to the actual size of a real MIDI keyboard. The audio was presented at approximately 65 dB pressure level through ER1-14B insert earphones via an HB7 Headphone Buffer (Tucker-Davis Technologies, US).

The audio and video presentation delay were recorded for all stimulus conditions (AO, VO, AV, and CO) with an audio-visual delay test toolbox connected with the EEG system (Electrical Geodesics, Oregon, US). Delay for the video signal, 57 ms (±2 ms jitter), and delay for the audio signal 50 ms (±12 ms jitter) were compensated later in the analysis.

Participants were instructed to relax and limit their eye movements during the experiment. The experiment included non-target trials (ca. 90%) and target trials (ca. 10%). A non-target trial included an AO, VO, AV, or CO stimulus and were the basis for later analyses. Non-target trials for the four conditions are illustrated in Figure 1. For example, a trial for the AV condition lasted 1036 ms (31 frames) and started with a 500 ms fixation cross at the location where the finger touched the C4 key. Afterward, a still image (first frame of the video) with a random interval [0, 100, 200] ms was presented before the video started. The first detectable finger movement frame (the video onset) started 165 ms before the auditory onset. Each trial ended with the last frame of the video, presented for 800 ms.

The role of target trials was to engage the participants, by pushing a button on a response pad 200 (Electrical Geodesics, USA), during the experiment. Since attention enhances activity in the sensory cortices corresponding to the modality of the stimulus, targets in each condition were the same modality as stimuli in that condition. As such, targets in the AO condition included a 120 ms beep (500 Hz) with two onset variations: 200 or 400 ms after the audio onset. Targets in the VO condition had a 120 ms white dot each time occurring above or below the C4 key, and targets in the AV condition was a synchronized beep (500 Hz) with a white dot (120 ms). Targets in the CO condition were a black dot (120 ms) on a gray background. Participants were asked to complete five practice trials to become familiar with the response box and experimental task.

For each of the four conditions, 246 non-target trials and 96 target trials were included, for a total of 1,080 trials pseudo-randomized across four blocks. In total the experiment took about an hour with a 3-min break after each block and 8 short pauses within each block.

### 2.4. EEG Recordings

Before the experimental session started, to select the best fit from the adult EGI EEG capsizes, the head size was measured for each participant based on their nasion-inion and preauricular distance. The cap was placed with Cz at the midpoint of the nasion.

EEG data were recorded using a 128-channel dense array EEG system connected to a Net Amps 300 amplifier (Electrical Geodesics, Oregon, US). Data were recorded at a 1,000 Hz sampling rate with no online filters, with Cz as the default reference electrode. Psychtoolbox (Pelli and Vision, 1997) and Net Station (5.2.0.2) were used for presenting the trials and recording the responses. Impedances were kept below 100Ω.

### 2.5. Data Analyses

Eeg recordings were interpolated to the 10-20 system (Jasper, 1958) and then imported into Matlab R2015b with EEGLAB (v15) extension (Delorme and Makeig, 2004). In EEGLAB a high-pass filter (0.5, 12 dB/octave) and a low-pass filter (48 Hz, 12 dB/octave) were applied and bad channels were removed. The remaining channels were re-referenced offline to the average reference. Independent component analysis was applied to remove stereotypical eye blinks, and EEG recordings were segmented starting 200 ms before and ending 500 ms after audio stimulus onsets (700 ms epochs).

Similar segmentation (from −200 to 500 ms), relative to the audio onset in the AO and AV trials, was applied for VO and CO trials. Baseline correction was performed from −200 to 0 ms. While multisensory effects are less evident for P1 (e.g., Stevenson et al., 2012), and P3 is sensitive to attention resources in an “odd ball” paradigm (Polich, 2007), N1-P2 reflects multisensory effects (e.g., Stekelenburg and Vroomen, 2007; Baart, 2016), and are attributed to previous musical experience (e.g., Shahin et al., 2003, 2005), and therefore was chosen for examining the AV interaction effect in each of the AV models. N1 was scored as the highest peak amplitude in a window of 75–120 ms and P2 in a window of 120–225 ms. Cz reflect activity originating from auditory-related brain regions (Bosnyak et al., 2004), has been vastly used in previous AV perception research (e.g., Baart, 2016), and therefore was chosen for further analyses. Based solely on non-target trials, to exclude the motor components due to responses in target trials (Luck, 2014), the average ERPs for each condition (AO, VO, AV, and CO) were calculated separately for musicians and non-musicians.

Previous research has shown that musicians have enhanced auditory processing which leads to increased N1 and P2 amplitudes (e.g., Shahin et al., 2003, 2005). Moreover, Baart (2016) suggested that the magnitude of amplitude and latency suppression of N1 and P2 in AV perception correlates with AO amplitudes and latencies. Therefore, the first analysis focused on the difference between musicians and non-musicians' N1 and P2 amplitudes and latencies in the AO condition.

For further analyses, to determine the effect of visual cues predicting the upcoming auditory signal in AV perception, compared to the auditory perception, four AV interaction models have been proposed. For model (1) (AV vs. AO+VO), AO and VO waveforms were added (AO+VO) to compare with the AV condition. For model (2) (AV-VO vs. AO), VO waveforms were subtracted from the AV waveforms (AV-VO) to remove the contribution of the visual signal from the ERPs for comparison with the AO condition. For model (3) (AV-VO vs. AO-CO), VO waveforms were subtracted from AV waveforms (AV-VO), and CO waveforms were subtracted from the AO waveforms (AO-CO) to compare auditory conditions. Finally, for model (4) (AV vs. AO), the AV waveforms were compared with AO waveforms.

First, for the AO condition, a one-way analysis of variance (ANOVA) was conducted with SPSS (v. 25) to examine statistical significance (α = 0.05) between musicians and non-musicians in AO N1 and P2 amplitudes and latencies. Then, each of the four AV models was evaluated in a two-way ANOVA with background (musicians vs. non-musicians) as a between-participant variable, component expression of the model (e.g., for model (1), AV and AO+VO) as a within-participant variable, and N1 and P2 amplitudes and latencies as dependent variables.

Note that, in the two-way ANOVA for each AV model, for the main effect of background, data from two modality conditions are collapsed (e.g., for model (1) AV averaged together with AO+VO). The main effect of background is therefore not a meaningful comparison between musicians and non-musicians and is not directly addressed. F and *p*-values are presented in Table 4 for assessment.

## 3. Results

As summarized in Table 2, musicians detected 95% of target trials on average, non-musicians detected 94% of the target trials indicating that during the experiment, both musicians and non-musicians similarly focused on the stimuli. Consistent with previous behavioral research (e.g., Strait et al., 2010) visual inspection of the means and variance for musicians, compared to non-musicians, showed a slightly higher percentage of correct responses in the detection task for the AO and AV conditions, however, the groups were similar in the VO and CO conditions.

**Table 2:** Percentage of correct responses with standard deviations in parentheses, for musicians and non-musicians in response to target trials in the audio only, video only, audiovisual, and control conditions.

	**Audio only (AO)**	**Video only (VO)**	**Audiovisual (AV)**	**Control (CO)**	**Average**
Musicians	95% (1)	95% (1)	96% (1)	94% (1)	95% (2)
Non-musicians	93% (1)	95% (1)	94% (1)	94% (1)	94% (3)

### 3.1. Audio-Only Condition

**Figure 2:**
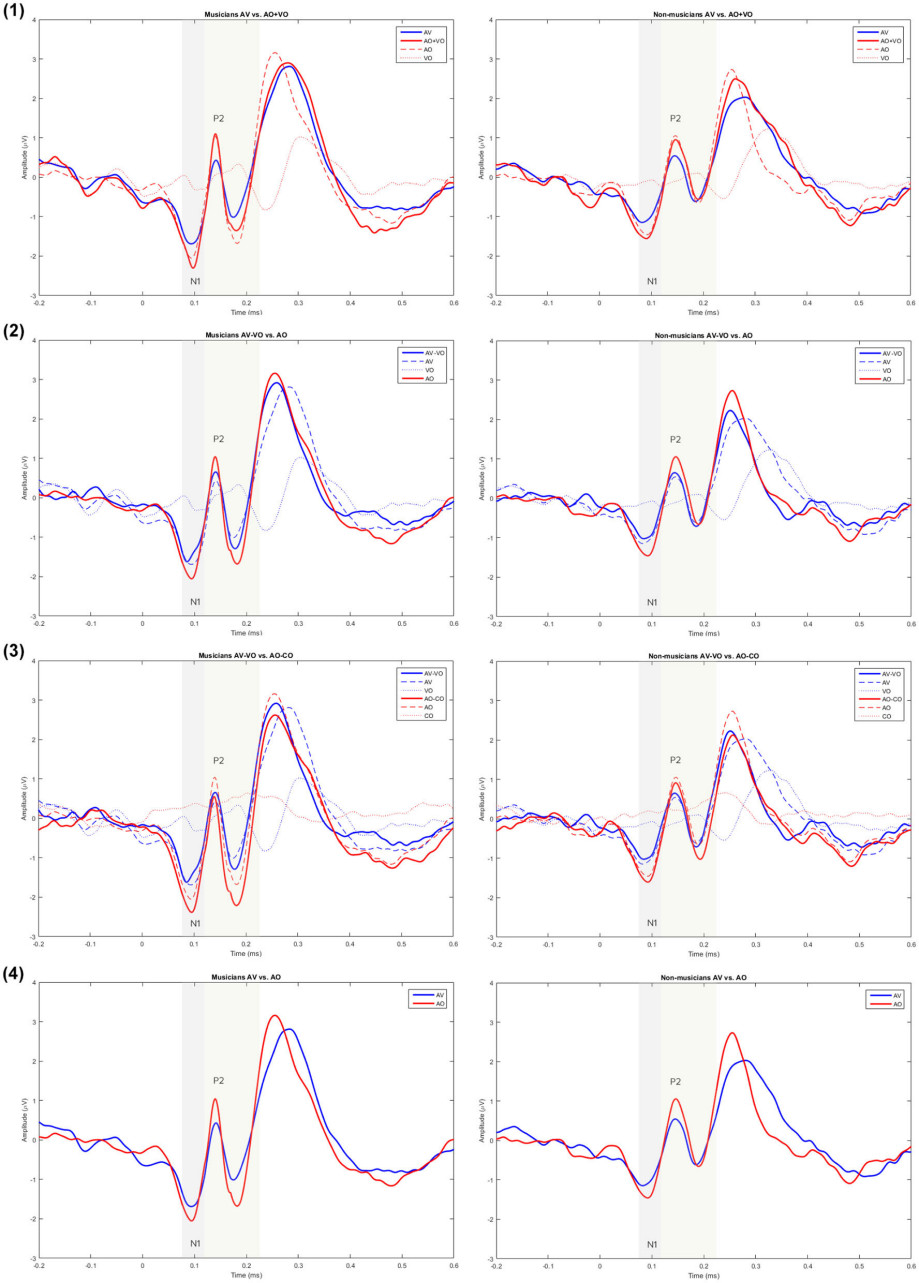
Grand averaged waveforms for musicians and non-musicians for model (1) AV vs. AO+VO, (2) AV–VO vs. AO, (3) AV–VO vs. AO–CO, and (4) AV vs. AO at Cz.

Musicians and non-musicians were compared based on their N1 and P2 amplitudes and latencies in the non-target trials in the AO condition (Figure 2). A one-way ANOVA for AO N1 amplitude showed a significant difference between musicians and non-musicians [*F*_(1, 38)_ = 5.25, *p* = 0.02], and as Table 3 shows, on average N1 was 0.55 μ*V* higher for musicians than for non-musicians. For the AO condition no group difference was observed for N1 latency [*F*_(1, 38)_ = 0.62, *p* = 0.43], P2 amplitude [*F*_(1, 38)_ = 0.07, *p* = 0.77], or P2 latency [*F*_(1, 38)_ = 3.2, *p* = 0.08].

**Table 3:** Means and standard deviations in parentheses for N1 and P2 amplitudes and latencies for musicians and non-musicians in the component expressions AO, AO-CO, AV, AV-VO, and AO+VO.

	**Component expression**	**N1 amplitude (μV)**	**N1 latency (ms)**	**P2 amplitude (μV)**	**P2 latency (ms)**
Musicians	AO	−2.20 (0.60)	96 (7)	1.48 (1.50)	142 (7)
	AO-CO	−2.54 (1.01)	97 (6)	1.11 (1.75)	141 (8)
	AV	−2.10 (1.18)	91 (14)	0.57 (1.09)	141 (6)
	AV-VO	−1.86 (0.87)	90 (12)	0.86 (0.86)	140 (5)
	AO+VO	−2.46 (0.78)	93 (13)	1.17 (1.78)	140 (5)
Non-musicians	AO	−1.65 (0.88)	93 (14)	1.38 (0.74)	147 (10)
	AO-CO	−1.80 (0.99)	88 (14)	1.21 (0.74)	150 (10)
	AV	−1.45 (0.90)	83 (18)	0.90 (0.81)	150 (14)
	AV-VO	−1.27 (0.77)	83 (15)	0.98 (0.89)	145 (11)
	AO+VO	−1.98 (1.18)	87 (18)	1.38 (1.02)	152 (13)

### 3.2. Audio-Visual Interaction

**Figure 3:**
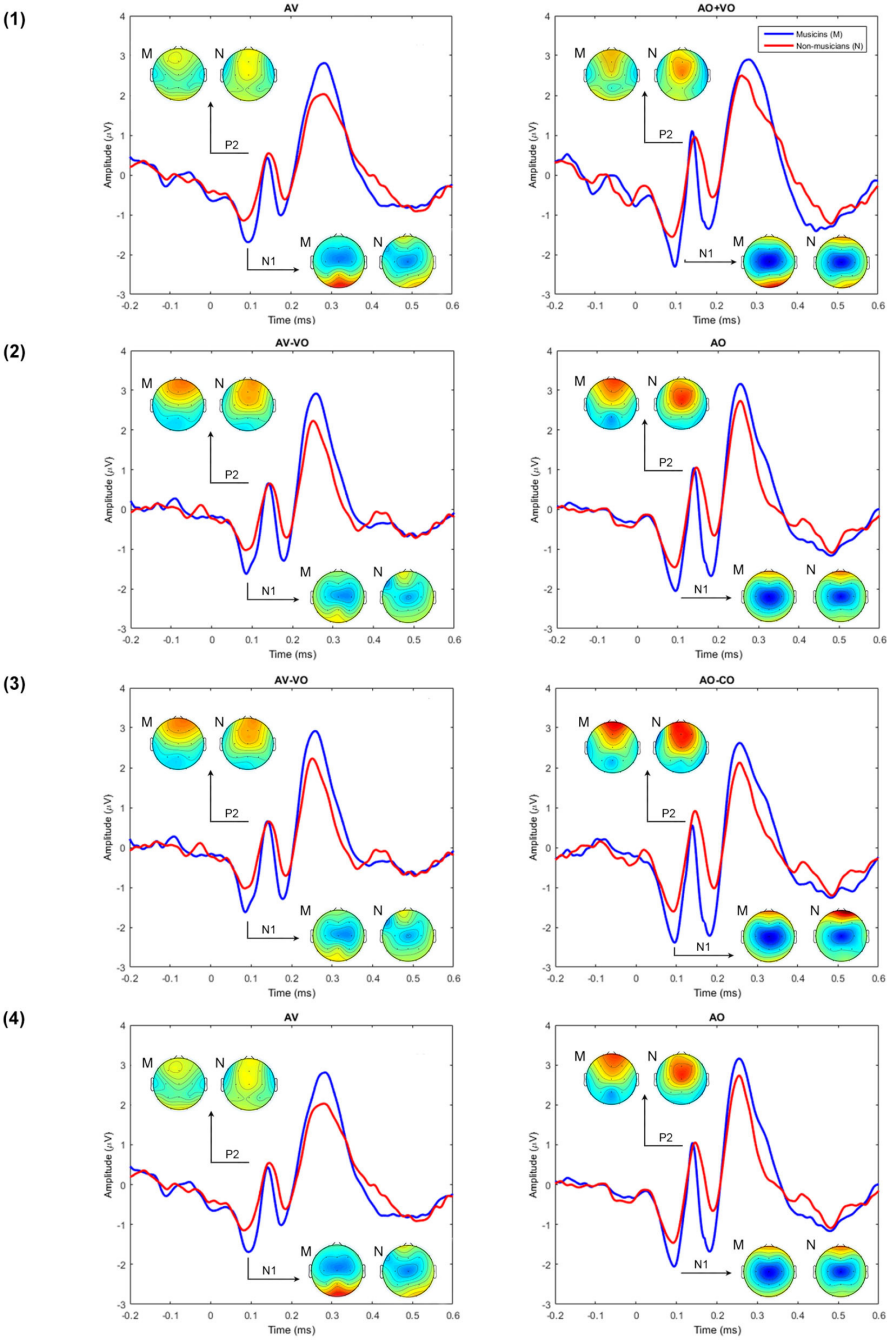
A comparison between musicians' (M) and non-musicians' (N) grand averaged waveforms at Cz for each of the AV interaction models **(1–4)** and the topographical maps at N1 and P2.

To compare musicians and non-musicians when the visual cues predict the upcoming musical audio signal, two-way ANOVAs were conducted based on non-target trials for each model, as Table 4 shows. Figures 2, 3 and Table 5 show the results of the four different models, for musicians and non-musicians.

**Table 4:** Summary of *F*-values for the four AV interaction models.

		**N1**		**P2**	
**AV model**		**Amplitude (μV)**	**Latency (ms)**	**Amplitude (μV)**	**Latency (ms)**
AV vs. AO+VO	Component expression	14.14^*^	0.95	13.08^*^	0.44
	Background	5.79^*^	3.61	0.92	18.44^**^
	Component expression × Background	0.49	0.11	0.10	1.55
AV-VO vs. AO	Component expression	11.15^*^	11.00^*^	19.82^**^	2.45
	Background	10.00^*^	7.52^*^	0.001	5.10^*^
	Component expression × Background	0.01	0.55	0.98	0.19
AV-VO vs. AO-CO	Component expression	23.65^**^	6.02^*^	2.23	2.76
	Background	9.62^*^	6.58^*^	0.21	9.12^*^
	Component expression × Background	0.43	0.16	0.002	1.89
AV vs. AO	Component expression	0.98	7.44^*^	22.38^**^	0.10
	Background	8.71^*^	2.56	0.17	8.01^*^
	Component expression × Background	0.08	0.67	2.01	0.51

*p ≤ 0.05 and **p < 0.0001.

**Table 5:** Summary of F-statistics, *p*-values, η^2^, and power for musicians and non-musicians.

**AV model**	**Group**	**ERP component**	**Measurement**	**F**	***p***	**η^2^**	**Power**
AV vs. AO+VO	Musicians	N1	Amplitude (μ*V*)	4.81	0.04	0.21	0.54
			Latency (ms)	0.24	0.62	0.01	0.07
		P2	Amplitude (μ*V*)	4.91	0.04	0.21	0.55
			Latency (ms)	0.37	0.54	0.01	0.08
	Non-Musicians	N1	Amplitude (μ*V*)	9.83	0.005	0.33	0.84
			Latency (ms)	0.78	0.39	0.03	0.13
		P2	Amplitude (μ*V*)	10.44	0.004	0.34	0.86
			Latency (ms)	1.27	0.27	0.06	0.18
AV-VO vs. AO	Musicians	N1	Amplitude (μ*V*)	5.81	0.02	0.24	0.62
			Latency (ms)	4.65	0.04	0.20	0.53
		P2	Amplitude (μ*V*)	9.97	0.005	0.35	0.84
			Latency (ms)	0.45	0.50	0.02	0.09
	Non-Musicians	N1	Amplitude (μ*V*)	5.56	0.02	0.21	0.61
			Latency (ms)	6.72	0.01	0.25	0.69
		P2	Amplitude (μ*V*)	10.06	0.005	0.33	0.85
			Latency (ms)	2.98	0.10	0.13	0.37
AV-VO vs. AO-CO	Musicians	N1	Amplitude (μ*V*)	16.72	0.001	0.48	0.97
			Latency (ms)	7.13	0.01	0.28	0.71
		P2	Amplitude (μ*V*)	0.65	0.43	0.03	0.11
			Latency (ms)	0.04	0.82	0.00	0.05
	Non-Musicians	N1	Amplitude (μ*V*)	8.31	0.009	0.29	0.78
			Latency (ms)	1.56	0.22	0.07	.220
		P2	Amplitude (μ*V*)	3.13	0.09	0.13	0.39
			Latency (ms)	4.10	0.05	0.17	0.48
AV vs. AO	Musicians	N1	Amplitude (μ*V*)	0.16	0.68	0.00	0.06
			Latency (ms)	2.30	0.14	0.11	0.30
		P2	Amplitude (μ*V*)	9.80	0.006	0.35	0.84
			Latency (ms)	0.18	0.67	0.01	0.06
	Non-Musicians	N1	Amplitude (μ*V*)	1.32	0.26	0.06	0.19
			Latency (ms)	5.42	0.03	0.21	0.60
		P2	Amplitude (μ*V*)	23.44	0.00009	0.54	0.99
			Latency (ms)	0.36	0.55	0.01	0.08

#### 3.2.1. Model (1): AV vs. AO+VO

As shown in Table 4, for the main effect of component expression, N1 amplitude $\left[F_{\left(1,\;38\right)}=14.14,p=0.001,\eta^{2}=0.27\right]$ was significantly lower for AV than AO+VO, however, for N1 latency $\left[F_{\left(1,\;38\right)}=0.95,p=0.33,\eta^{2}=0.02\right]$ results showed no significant difference between AV and AO+VO. Moreover, while P2 amplitude $\left[F_{\left(1,38\right)}=13.08,p=0.001,\eta^{2}=0.25\right]$ was lower for AV than for AO+VO, P2 latency $\left[F_{\left(1,\;38\right)}=0.44,p=0.5,\eta^{2}=0.01\right]$ showed no significant difference between the two component expressions.

No significant interaction was observed between the effect of component expression and background for N1 amplitude $\left[F_{\left(1,\;38\right)}=0.48,p=0.49,\eta^{2}=0.01\right]$, N1 latency $\left[F_{\left(1,\;38\right)}=0.11,p=0.73,\eta^{2}=0.003\right]$, P2 amplitude $\left[F_{\left(1,\;38\right)}=0.1,p=0.74,\eta^{2}=0.003\right]$, or P2 latency $\left[F_{\left(1,\;38\right)}=1.55,p=0.22,\eta^{2}\;=\;0.03\right]$.

#### 3.2.2. Model (2): AV-VO vs. AO

Results (Table 4) from the main effect of component expression showed lower N1 amplitude $\left[F_{\left(1,38\right)}=11.15,p=0.002,\eta^{2}=0.22\right]$, N1 latency, $\left[F_{\left(1,\;38\right)}=11,p=0.002,\eta^{2}=0.22\right]$, and P2 amplitude $\left[F_{\left(1,\;38\right)}=19.82,p=0.00007,\eta^{2}=0.34\right]$ in AV-VO compared to the AO. However, the results for P2 latency $\left[F_{\left(1,\;38\right)}=2.45,p=0.12,\eta^{2}=0.06\right]$ showed no significant difference between the two component expressions.

Furthermore, the results (Table 4) showed no significant interaction between the effect of component expression and background for N1 amplitude $\left[F_{\left(1,\;38\right)}=0.01,p=0.89,\eta^{2}=0.0004\right]$, N1 latency $\left[F_{\left(1,\;38\right)}=0.55,p=0.46,\eta^{2}=0.01\right]$, P2 amplitude $\left[F_{\left(1,\;38\right)}=0.98,p=0.32,\eta^{2}=0.02\right]$, and P2 latency $\left[F_{\left(1,\;38\right)}=0.19,p=0.66,\eta^{2}=0.005\right]$.

To investigate if AO N1 amplitude, which was enhanced in musicians, contributes to the N1 suppression effect in AV-VO, a Pearson correlation coefficient was computed and showed a significant correlation between AO N1 amplitudes and AV-VO N1 amplitudes for musicians [*r* = 0.70, *n* = 19, *p* = 0.001], and non-musicians [*r* = 0.62, *n* = 21, *p* = 0.002].

#### 3.2.3. Model (3): AV-VO vs. AO-CO

Results (Table 4) showed lower N1 amplitude $\left[F_{\left(1,\;38\right)}=23.65,p=0.00002,\eta^{2}=0.38\right]$, N1 latency $\left[F_{\left(1,\;38\right)}=6.02,p=0.01,\eta^{2}=0.13\right]$, in AV-VO compared to AO-CO, however, results from P2 amplitude $\left[F_{\left(1,\;38\right)}=2.23,p=0.14,\eta^{2}=0.05\right]$, and P2 latency $\left[F_{\left(1,\;38\right)}=2.76,p=0.1,\eta^{2}=0.06\right]$ showed no significant difference between the two component expression.

Furthermore, the interaction (Table 4) between the effect of component expression and background was not significant for N1 amplitude $\left[F_{\left(1,\;38\right)}=0.43,p=0.51,\eta^{2}=0.01\right]$, N1 latency $\left[F_{\left(1,\;38\right)}=0.16,p=0.68,\eta^{2}=0.004\right]$, P2 amplitude $\left[F_{\left(1,\;38\right)}=0.002,p=0.96,\eta^{2}=0.00004\right]$, and P2 latency $\left[F_{\left(1,\;38\right)}=1.89,p=0.17,\eta^{2}=0.04\right]$.

#### 3.2.4. Model (4): AV vs. AO

While the results (Table 4) from N1 amplitude $\left[F_{\left(1,\;38\right)}=0.98,p=0.32,\eta^{2}=0.02\right]$ showed no significant difference between AV and AO component expression, results showed lower N1 latency $\left[F_{\left(1,\;38\right)}=7.44,p=0.01,\eta^{2}=0.16\right]$, and P2 amplitude $\left[F_{\left(1,\;38\right)}=22.38,p=0.00003,\eta^{2}=0.37\right]$ in AV compared to the AO component expression. P2 latency $\left[F_{\left(1,38\right)}=0.1,p=0.75,\eta^{2}=0.003\right]$ results showed no significant difference between component expressions either.

Moreover, the results (Table 4) showed no significant interaction between the effect of component expression and background for N1 amplitude $\left[F_{\left(1,\;38\right)}=0.08,p=0.76,\eta^{2}=0.002\right]$, N1 latency $\left[F_{\left(1,\;38\right)}=0.67,p=0.41,\eta^{2}=0.01\right]$, P2 amplitude $\left[F_{\left(1,\;38\right)}=2.01,p=0.16,\eta^{2}=0.05\right]$, or P2 latency $\left[F_{\left(1,\;38\right)}=0.51,p=0.47,\eta^{2}=0.01\right]$.

In summary, in auditory perception musicians showed a higher N1 amplitude than non-musicians. In AV perception the pattern of results for musicians and non-musicians was consistent for each of the four models, however, N1 and P2 results varied across the models. For model (1), both N1 and P2 amplitudes were lower in AV compared to AO+VO. In model (2), N1 amplitude and latency and P2 amplitude were lower in AV-VO compared to AO, P2 latency did not show a significant difference. In model (3), N1 amplitude and latency were lower in AV-VO compared to AO-CO, while P2 amplitude and latency showed no difference. In model (4), N1 latency and P2 amplitude were lower in AV compared to AO, while N1 amplitude and P2 latency did not show this pattern.

## 4. Discussion

The current study first aimed to examine the expression of AV interaction models through N1 and P2 in AV music perception, based on the visual finger and hand movements predicting audio music. Results showed that calculation differences across AV interaction models did not lead to the same pattern of results for N1 and P2 amplitude and latency suppression. Previous research (Baart, 2016) suggested that AV interaction at N1 and P2 amplitudes and latencies are positively correlated with the magnitude of N1 and P2 amplitudes and latencies in auditory music. To test this, musicians and non-musicians were first compared based on N1 and P2 amplitudes and latencies in response to auditory music. Then, for each of the four AV interaction models (1), (2), (3), (4), musicians and non-musicians were compared based on N1 and P2 amplitudes and latencies, to examine if potentially enhanced auditory N1 and P2 amplitudes in musicians leads to more suppression of N1 and P2 amplitudes and latencies in AV perception. Although musicians, compared to non-musicians, showed higher N1 amplitude in auditory music perception, no difference was found between the two groups in any of the AV interaction models.

### 4.1. Comparison of AV Interaction Models

Visual information which is naturally starting before the auditory signal can provide visual cues and form a prediction about the upcoming corresponding auditory signal. This prediction leads to a decrease in N1 and P2 amplitudes and latencies, both with speech (Besle et al., 2004; Van Wassenhove et al., 2005; Arnal et al., 2009; Pilling, 2009; Baart et al., 2014; Baart, 2016; Paris et al., 2016b), and non-speech stimuli (Vroomen and Stekelenburg, 2010; Stekelenburg and Vroomen, 2012; Paris et al., 2017). In line with these studies, here, in AV, when the visual cues from finger and hand movements predict the upcoming musical sound, compared to the auditory music perception, N1 amplitudes decreased in models (1), (2), (3), and P2 amplitudes decreased in model (1), (2), and (4).

Previous research (Van Wassenhove et al., 2005; Stekelenburg and Vroomen, 2007, 2012; Arnal et al., 2009; Paris et al., 2016b, 2017) showed that N1 and P2 amplitude suppression in AV perception have different underlying processes. N1 is modulated when the visual cues predict the upcoming sound and is sensitive to visual predictability (Arnal et al., 2009; Paris et al., 2017), and spatial properties (Stekelenburg and Vroomen, 2012). However, P2 suppression reflects the AV content congruency between the visual information and perceived auditory signal (Van Wassenhove et al., 2005; Arnal et al., 2009; Paris et al., 2016b). Moreover, previous research (Stekelenburg and Vroomen, 2007; Baart, 2016) suggested that based on model (3) (AV-VO vs. AO-CO) with non-speech stimuli, P2 amplitude might show more suppression than N1 amplitude. Moreover, Baart (2016) examined AV interaction at N1 and P2 amplitudes with two models (AV vs. AO, and AV-VO vs. AO) and also suggested that while comparing AV and AO perception (AV vs. AO) showed no difference between the amplitude at N1 and P2, when comparing the AO with the visual waveform subtracted from AV (AV-VO vs. AO), amplitude suppression was smaller for N1 than P2. Baart (2016) argued that the difference in amplitude suppression between N1 and P2 was a result of the inclusion or exclusion of particular data in the meta-analysis.

In line with these studies, the current results also showed that AV interaction models do not express N1 and P2 amplitude suppression in AV perception in the same way. Superposition of waves leads to calculation differences across the four AV interaction models for N1 and P2 amplitudes. As Figure 2 shows, model (1) (AV vs. AO+VO) led to a suppression for both N1 and P2 amplitudes in AV interaction. The difference waves in model (2) and (3) both reflect audio waveforms and use the same component expression but nevertheless led to inverse suppression magnitude for N1 and P2 amplitudes; the difference lies in their second component expressions. In model (2) (AV-VO vs. AO), subtracting the visual evoked signal from the AV evoked signal (AV-VO) led to a higher N1 amplitude than for AO N1 amplitude and a smaller AV-VO P2 amplitude than AO P2 amplitude. Therefore, model (2) expressed less AV amplitude suppression for N1 than P2. In model (3), subtracting the CO evoked signal, which does not have typical ERP components, from the AO evoked signal led to a slightly higher AO-CO N1 amplitude than AV-VO N1 amplitude, and a slightly lower AO-CO P2 amplitude than AV-VO P2 amplitude. Therefore, these calculations in model (3) led to less AV amplitude suppression for P2 (which did not show significant suppression) than N1. In model (4) (AV vs. VO), when comparing N1 and P2 amplitudes in AV and AO, while N1 amplitude showed no significant difference between AV and AO perception, AV P2 is suppressed compared to the AO P2 amplitude. These findings suggest that the calculation differences across four AV interaction models lead to different results for N1 and P2 amplitudes.

All AV interaction models except model (1), in which N1 latency was non-significantly lower in AV compared to the summation of auditory and visual waveforms, expressed N1 latency suppression in AV perception. While what lies behind model (1) N1 latency results can only be speculated, this finding is in line with Pilling (2009) in which, by applying model (1), no significant decrease had been found for N1 and P2 latencies in AV, compared to AO+VO. On the other hand, none of the four AV interaction models led to P2 latency suppression in AV perception, compared to the auditory perception (AO or AO-CO). A meta-analysis (Baart, 2016) of AV speech perception showed that most of the studies found lower P2 latency in AV compared to the auditory perception, however, not having a latency decrease for P2 is not uncommon (e.g., Pilling, 2009; Baart et al., 2014). Furthermore, while in contrast with the current results of music, Stekelenburg and Vroomen (2007) showed P2 latency suppression for non-speech stimuli in AV perception, and Paris et al. (2017) did not report any P2 latency suppression with non-speech-stimuli. Therefore, although generally, AV interaction models express lower P2 latency in AV perception than auditory perception, having no latency decrease in AV perception has been reported with speech and non-speech stimuli.

Across the models, the current results from model (2) were most consistent with previous findings, expressing N1 amplitude and latency and P2 amplitude suppression in AV perception. Previous research generally showed that N1 and P2 amplitudes and latencies are lower in AV perception due to predictive movements starting before the corresponding auditory signal, compared to auditory perception. Although comparing auditory and AV perception began with model (1), most research on AV speech and non-speech perception used model (2) (e.g., Stekelenburg and Vroomen, 2007; Baart, 2016). In line with previous findings, the current results from model (2) were more consistent with previous research and expressed N1 amplitude and latency and P2 amplitude suppression in AV perception.

### 4.2. Musicians and Non-musicians

Previous research has suggested that musicians have enhanced auditory music perception compared to non-musicians (Pantev et al., 2001; Shahin et al., 2003, 2005; Kuriki et al., 2006; Baumann et al., 2008; Virtala et al., 2014; Maslennikova et al., 2015; Rigoulot et al., 2015, for a review see, Sanju and Kumar, 2016, but also see, Lütkenhöner et al., 2006). Current results showed that musicians, compared to non-musicians, had higher N1 amplitude in auditory music. P2 amplitude was not significantly different between musicians and non-musicians, even though P2 amplitude mean for musicians was slightly higher. In line with current results, Baumann et al. (2008) compared musicians and non-musicians based on their N1 and P2 amplitudes in response to auditory music stimuli and showed that musicians have increased N1 amplitude, however, they found no difference between the two groups for P2 amplitude. The intrinsic functional relevance of N1 enhanced amplitude for musicians, compared to non-musicians, is still unclear (e.g., Kühnis et al., 2014). Previous research has shown that musicians' enhanced N1 amplitude was associated with their better performance in a discrimination task (Virtala et al., 2014), and enhanced intracerebral beta oscillation, which is involved in sensory processing (e.g., Haenschel et al., 2000), predictive timing (Doelling and Poeppel, 2015), attentional shift (van Ede et al., 2014), and auditory-motor interactions (Large and Snyder, 2009). Moreover, Shahin et al. (2003) showed that musicians, compared to non-musicians, have a higher P2 amplitude in response to auditory music stimuli. In their later study (Shahin et al., 2005), they showed that musicians have higher P2 and N1 amplitude, even though the results for N1 amplitude were not significant, compared to non-musicians. Although results differ in previous research comparing musicians and non-musicians' N1 and P2 amplitudes in response to music stimuli, research generally has shown that musicians have enhanced amplitudes at both N1 and P2 or at least one of the components due to their previous musical training.

Based on the suggestion by Baart (2016) that the magnitudes of N1 and P2 amplitudes and latencies are positively correlated between AO and AV perception, the difference in suppression of N1 and P2 between musicians and non-musicians in auditory music perception could be expected to be correlated with a corresponding group difference in AV music perception. Specifically, for AV music perception, greater suppression at N1 and P2 would be expected for musicians compared to non-musicians. Current results for music stimuli showed that while both musicians and non-musicians showed a positive correlation between auditory and AV perception (AO and AV-VO), no difference was found between musicians and non-musicians applying any of the AV interaction models. These results for music stimuli imply that musicians' enhanced auditory perception, compared to non-musicians, does not necessarily lead to enhanced AV perception.

The question then arising from the current results is why musicians did not show enhanced suppression in early components for AV music perception, when they had enhanced auditory perception. In a study (Sorati and Behne, 2019) with the same group of participants and using speech materials with model (2) (AV-VO vs. AO), musicians showed more N1 amplitude suppression than non-musicians. The difference between the current N1 amplitude findings and those in Sorati and Behne (2019) is plausibly explained by the use of music stimuli vs. speech stimuli. Moreover, Stekelenburg and Vroomen (2007) have suggested that N1 and P2 amplitude and latency suppression in response to AV non-speech stimuli is more than speech stimuli, since the predictability of visual cues before the auditory sound in non-speech stimuli, such as finger and hand movements when playing a musical instrument, might be more than the predictability of facial articulatory movements in AV speech. Therefore, an explanation for musicians and non-musicians' different results in response to speech and music stimuli might be that for music stimuli, due to more predictability of finger and hand movements, both musicians and non-musicians predict the upcoming musical sound similarly. However, for AV speech stimuli, as the articulatory movements were not as explicit as finger and hand movements in AV music, only musicians showed N1 amplitude suppression in AV speech perception.

Findings for model (2) in the current study are based on Sorati and Behne (2020) where, in addition to ERP analyses, inter-trial phase coherence (ITPC) was also evaluated. ITPC measures oscillatory activity which in low-frequency bands (<30 Hz) influence forging of early evoked potentials such as N1 and P2 (e.g., Edwards et al., 2009). Sorati and Behne (2020) showed that in response to music, ITPC differed for musicians and non-musicians indicating differences (e.g., sensory-motor processing) in their underlying mechanisms. These results indicate that although previous studies (e.g., Sorati and Behne, 2020) showed underlying differences between musicians and non-musicians, these differences did not come through in the ERP results based on any of the four AV interaction models.

## 5. Conclusions

Previous research has led to four AV models to examine the interaction of auditory and visual perception when the visual cues predict an upcoming auditory signal and lead to N1 and P2 suppression. However, a meta-analysis (Baart, 2016) suggested that AV perception does not always lead to N1 and P2 suppression. To examine variability in N1 and P2 across AV perception research, the current study first aimed to determine the influence of four AV interaction models extensively applied in previous research on N1 and P2 suppression in AV music perception. The current findings for AV music perception indicate that the calculation differences across the models lead to differences in the expressions of AV interaction through N1 and P2 amplitude and latency, as well as inverse suppression magnitudes for N1 and P2.

The current study also tested the suggestion (Baart, 2016) that the N1 and P2 suppression in AV and auditory perception are positively correlated by comparing musicians and non-musicians. In line with previous research (e.g., Baumann et al., 2008), the current findings showed that in auditory music perception, musicians, compared to non-musicians, have enhanced N1 amplitude, however, in AV perception and similarly in all of AV interaction models, musicians and non-musicians showed similar N1 and P2 amplitudes and latencies suppression. These findings indicate that when the visual cues from finger and hand movements predict the upcoming musical sound in AV music perception, musicians and non-musicians, regardless of musicians' enhanced auditory perception, show similar suppression for early evoked potentials. These ERP results did not reflect the difference between musicians and non-musicians in underlying mechanisms such as sensory-motor processing.

This study also highlights the effect of four AV interaction models on early evoked potentials and indicates that due to the calculation differences across models they do not leave the same pattern of results for N1 and P2. These results also indicate that the four AV interaction models are not interchangeable and are not directly comparable.

## Data Availability Statement

The raw data supporting the conclusions of this article will be made available by the authors, without undue reservation.

## Ethics Statement

This study, involving human participants, was reviewed and approved by Norwegian Center for Research Data (NSD). Participants provided their written informed consent to participate in the study.

## Author Contributions

Both authors contributed extensively to the work presented in this paper. MS and DB jointly conceived of the study and sketched the design. MS carried out the practical implementation of the project, carried out the EEG experiments and data analyses, and drafted the full paper. DB supervised all stages of the project. Both authors discussed the results and implications and contributed to the manuscript.

## Conflict of Interest

The authors declare that the research was conducted in the absence of any commercial or financial relationships that could be construed as a potential conflict of interest.
